# Strictures and Inversions of the Uterus by Dr. White

**Published:** 1838

**Authors:** M. Z. Kreider

**Affiliations:** Corresponding Sec’y of said Association; Lancaster, Ohio


### Art. III.—Strictures of the Uterus.

*A letter to one of the Editors containing practical remards on the treatment of inversions and strictures of the Uterus*—By M. Z. Kreider, M. D.

Lancaster, Ohio, May 18, 1838.

*Dear Doctor:*

Being aware of the lively interest you feel in relation to the advance of Medical and Physical Science over the world in *general*, and the *Western* country in *particular*, I have thought proper, however humble the source, and unpretending the effort, to advise you of a recent movement of some of the physicians in Fairfield county. Believing as we do, that it is the imperative duty of every practitioner of the healing art, to contribute his mite to the general stock of knowledge, and being aware that this could not be so advantageously done without concert and harmony of action, a respectable number of the physicians of this county resolved to form themselves into an Independent Medical Society, for the purpose of rendering mutual aid, and of elevating the character of the profession in this community. To this end, a meeting was held on the 22d of February last, and a Society was regularly organized, entitled, "The Fairfield County Medical Association for the cultivation and improvement of the Medical and Physical Sciences." None are admitted to membership in this Society, unless they sustain a good moral character, and have studied, and been admitted, in a regular manner. There are four stated meetings in each year, and each member is required to report from time to time, all important cases which occur in practice, and essays are required to be read at every meeting, important questions are also required to be proposed and debated. At a recent meeting three essays were read, one by Doctor J. White, on the propriety of dividing the stricture in strangulated uterus, one by Doctor Bigelow, on the importance of the study of Botany to the Physician; and one by Doctor Kreider, on the history, nature, symptoms and cure of Variola, Varioloid and Vaccina.—The particular reason which induced me to select this subject was the prevalence of Small Pox among us at the time, and the little experience of the junior members of the profession in regard to its nature and treatment. The Society has created standing committees on Botany and Meteorology. The members of which reside in various parts of the county, and are required to collect botonical specimens, and note meteorological observations, and forward the same to *Reporters* located in Lancaster, whose duty it is to properly arrange the matter thus acquired, and report the same quarterly to the Society.

As the paper read by my friend Doctor White, proposes a novel remedy for stricture of the uterus, and as it is desirable that the method he has proposed should have publicity, to the end that its efficacy may be tested, I have thought proper to subjoin an extract from his paper which I hope may not be deemed uninteresting.

"In a practice of sixteen years I do not recollect to have met with a single case of inversion of the uterus. Though, I have heard of three cases, one of which occurred in the practice of an intelligent physician; the other two was under the care of female accoucheurs, the former was immediately reduced and without difficulty, the latter, were instances of complete inversion of that organ, brought about by rudeness and violence in the extraction of the placenta. It is a matter of some surprise that accidents of this terrible nature do not occur more frequently, when we consider the number of individuals in parturition who confide the management of their cases into the hands of the ignorant. It is not uncommon, with many of those practitioners when the placenta does not follow the fœtus with that celerity consistent with their ideas of those matters, to trust less to the physiological laws of the economy governing this important process, than to their own manipulations. Apparent difficulties with them, are too frequently construed into a warrant for extraordinary and mischievous exertion, and the consequence is, we fear more frequently than is generally known, the accident now under consideration.

The reasons why accidents of this kind are not of common occurrence I conceive to be the following:—Inversion of the uterus cannot well take place when it is in a state of general contraction, it is only when it contracts irregularly, that we have fears of this accident from improper manouvres. It must be in a state of general relaxation, or the fundus and body in this condition. Now, it is a well ascertained fact, that traction by the cord, excites the tonic contraction of the uterus; and it is commonly alone depended on by the ignorant to obtain the expulsion of the placenta. In addition, tractions made in a line with the inferior trait, and consequently at a considerable angle with the axis of the brim are comparatively little calculated to do mischief. Inversion sometimes occurs after the extraction of the placenta, and occasionally, some days after parturition, from some peculiarity in the condition of the uterus, not well understood, giving a predisposition thereto. Dr. Dewees, therefore infers, that pulling by the funis is not generally, as was believed, the cause of this accident. A case occurred to me in 1834, which seems to corroborate the Doctor's opinion. Mrs. H. in labour with her first child, suffered very much from slow and tedious dilitation of the os tincæ, the breech presented, in the first position of Baudeloque, nothing of consequence occurred until the head descended into the cavity of the pelvis, the arms were brought down without difficulty, but the head became fixed in its diagonal position, though the pains were strong, the head was ascertained to be of medium size, and the pelvis ample. Turning the face into the hollow of the sacrum and depressing the chin together with pretty strong traction did not suffice as I had anticipated, to bring down the head. I could not account for the difficulty. But on renewing my efforts, and exerting all the force I dared exert, (the fœtus being dead) it suddenly came to hand, the resistance giving way with a considerable jirk. The cord was cut, and the fœtus wrapped up in a cloth under the bedclothes, and handed to an assistant without examination. I then undertook the delivery of the placenta; astonished at finding no funis; and still more so when examining per vaginam no placenta, I immediately examined the child, and found the placenta attached to the scalp over the vertex of the head, and a short cord descending from it to the umbilicus. Fearing an inversion of the uterus from the strong traction employed, I immediately ascertained its condition, and found it contracted and well placed.

I remember having been called to the assistance of a poor woman, whose child was born before my arrival. Getting out of bed in obedience to a certain irritation of the rectum, the child was suddenly expelled, fell on the floor and ruptured the funis within an inch and a half of the umbilicus, there was no inversion of the uterus."

After recapitulating the varieties and symptoms of inversion of the uterus, the Doctor thus proceeds—

"But from all the evidence we can gather in the cases, it appears that the chief danger results—First, from the condition of the uterus; and secondly, from hæmorrhage, either previously existing, or super-induced by the abnormal position of the uterus. The latter however, existing in the relation of an effect only, we must look to the condition of the uterus in order to ascertain the real cause of existing difficulties, or to deduce curative indications. The condition of the uterus therefore, I conceive, constitutes the principal source of danger, and that danger is to be estimated by the intensity of the stricture formed by the contraction (temporary or permanent) of the os tincæ or cervix uteri. Happily for the unfortunate subjects of this serious accident, it is as we are taught to believe, amenable to the resources of art, if the inversion be discovered early and the most approved remediate means are immediately and perseveringly employed. But if from neglect, or from misconception of the symptoms announcing the inversion, the proper time for action be lost, the patient may get into a state of great danger, resulting from irregular contraction, producing strangulation of the uterus. When the os tincæ, and cervix uteri contract firmly upon the body or fundus of the uterus the most alarming symptoms result. Vomiting, great anxiety, pain, faintness, cold sweats, a scarcely perceptible or extinct pulse, delirium, convulsions, &c., all in a rapid succession, and if not soon relieved terminating in death. Here, then we have no time for delay, no time to temporize in the vain hope that the stricture will relax, the uterus soften, and in a day or two present a favorable condition for reduction. Nor is it proper to consume valuable time, to torture the poor patient in persisting in fruitless and unsuccessful attempts to force the uterus through a rigid aperture, that bears no proportion to its magnitude, adding thereby to the danger, as well as the sufferings of the unfortunate woman. Delay on the contrary is necessary, nay imperative! when the stricture is not severe, and the symptoms not threatening, since we have histories of cases of successful reduction several days after the occurrence of the accident. After adverting to the plan adopted by Dewees of rendering relief by pulling down the uterus in incomplete inversion, he proceeds—"In my opinion, (and it is a mere opinion only, since it never has been practically demonstrated) the indications in strangulated uterus, are the same as in strangulated hernia, venesection, tobacco, &c., excepted. The lesion of function and of structure are very nearly analagous. In strangulated hernia, we have precisely the same train of symptoms, save the hæmorrhage, &c., that occur in strangulated uterus, viz: anxiety, nausea, vomiting, pallor, faintness, cold sweats, failure of the pulse, &c. These symptoms proceed from the stricture made upon the contents of the hernial sac. What are the local results of stricture of those parts? Disturbed function, inflammation, and ultimately gangrene. The same events sometimes follow strangulation of the uterus, *to wit*, inflammation and sloughing of that organ. Dividing the stricture and placing the protruded parts in situ, would relieve existing difficulties, both general and local in either case. And it is my deliberate opinion, that the division of the stricture formed either by the os tincæ or cervix uteri, in the cases of strangulated uterus under consideration, is as fully indicated, as it is in incarcerated hernia. Why should not the stricture girding the uterus be divided, as well as one embracing the omentum, or the intestines? Are the symptoms less threatening, or is the accident less fatal? Would the division of the stricture in the one case be more impracticable or hazardous than in the other? I think not. The os tincæ and cervix uteri have been divided again and again without involving consequences more serious than those occuring occasionally from division of the stricture in hernia. And I believe the demand for a division of the stricture in the one case is fully as imperative, as it is in the other."

I cannot proceed in the brief space of a letter to recapitulate the features of the other essays read before the association, but hope to be able hereafter to advise you of our transactions. In conclusion, allow me to add, that I regret the apparent apathy that obtains too widely among our medical brethren in the west, in relation to the adoption of efficient measures for the promotion, culture and improvement of our noble science. Among the most feasible, ready and certain means of obtaining this desirable end, I believe the voluntary associations of its votaries stands prominent. In relation to the importance and bearing such societies have, our President well remarked. "To what causes shall we ascribe the elevation to which the profession of medicine has attained at the present day, ranging it by the side of the *certain* sciences—those of physic, geometry, chemistry &c. Is it due to the ancient and once universally accredited notion of vitiated fluids, the ingenious and captivating theories of Stahl, of Hoffman, of Cullen, of Brown, of Darwin, of Rush, and a hundred others, equally vague and visionary. I fearlessly answer no! We owe it to medical institutions and societies. And who do we find at the head of those institutions and societies? The great and distinguished cultivators of organic and physiological medicine. The vain imaginings of the *theorists* have fallen before the ordeal of a rigid philosophy, a careful analysis and sound induction."

This hasty and imperfect letter, you are hereby authorized to use as your judgment may dictate. If deemed of sufficient interest to merit a place in your valuable Journal, we shall be well pleased, if not, do with it as you like, I shall be satisfied.

I remain dear sir, very truly yours &c.

M. Z. KREIDER.

*Corresponding Sec'y of said Association.*

